# Supplementary material for: Parallel and convergent genomic changes underlie independent subterranean colonization across beetles
Source: Nat Commun. 2023 Jun 29;14:3842. doi: 10.1038/s41467-023-39603-1 (PMC10310748; doi:10.1038/s41467-023-39603-1)
Supplement: Supplementary file 1 — Supplementary Information [file 41467_2023_39603_MOESM1_ESM.pdf]

Supplementary Information

Parallel and convergent genomic changes underlying independent subterranean colonization across beetles
Genomic exaptation and convergent evolution paved the way to independent subterranean colonization across beetle lineages

Pau Balart-García[1*], Leandro Aristide[1], Tessa M. Bradford[2,3], Perry G. Beasley-Hall[2,3], Slavko Polak[4], Steven J. B. Cooper[2,3], Rosa Fernández[1*]

[1] Metazoa Phylogenomics Lab, Biodiversity Program, Institute of Evolutionary Biology (CSIC - Universitat Pompeu Fabra). Passeig Marítim de la Barceloneta 37-49, 08003 Barcelona, Spain
[2] Department of Ecology and Evolutionary Biology, School of Biological Sciences, and Environment Institute, University of Adelaide, Adelaide, South Australia 5005, Australia
[3] South Australian Museum, Adelaide, South Australia 5000, Australia
[4] Notranjska Museum Postojna, Kolodvorska c. 3, 6230 Postojna, Slovenia

*Corresponding authors: pau.balart@ibe.upf-csic.es, rosa.fernandez@ibe.upf-csic.es

Supplementary Tables

Supplementary Table 1. Fossil calibration points used for the divergence time estimation with MCMCtree. Codes indicated correspond to those of Figure 1.

**Split codes**	**Fossil**	**Age (Mya)**	**References**
(1) Root Age - Holometabola	*Srokalarva berthei*	358.9	1
(2) Crown Coleoptera	*Ponomarenkium belmonthensis*	307.1 - 251.878	2
(3) Coleoptera - Archostemata	*Kirghizocupes proporeius*	235 - 221.5	1
(4) Coleoptera - Adephaga	*Tunguskagyrus planus*	254.2 - 252.2	1
(5) Coleoptera - Curculionoidea	*Archaeorrhynchus* and/or *Eobelus*	166,1 - 157,3	1
(6) Crown Polyphaga	Undescr. taxon of the series Elateriformia	293.69 - 237	2
(7) Derodontidae - Clambidae	*Juropeltastica sinica*	251.878 - 165	2

1. McKenna, D. D. *et al.* The evolution and genomic basis of beetle diversity. *Proc. Natl. Acad. Sci. U. S. A.* **116**, 24729–24737 (2019).
2. Cai, C. et al. Integrated phylogenomics and fossil data illuminate the evolution of beetles. *R Soc Open Sci* **9**, 211771 (2022).

Supplementary Figures

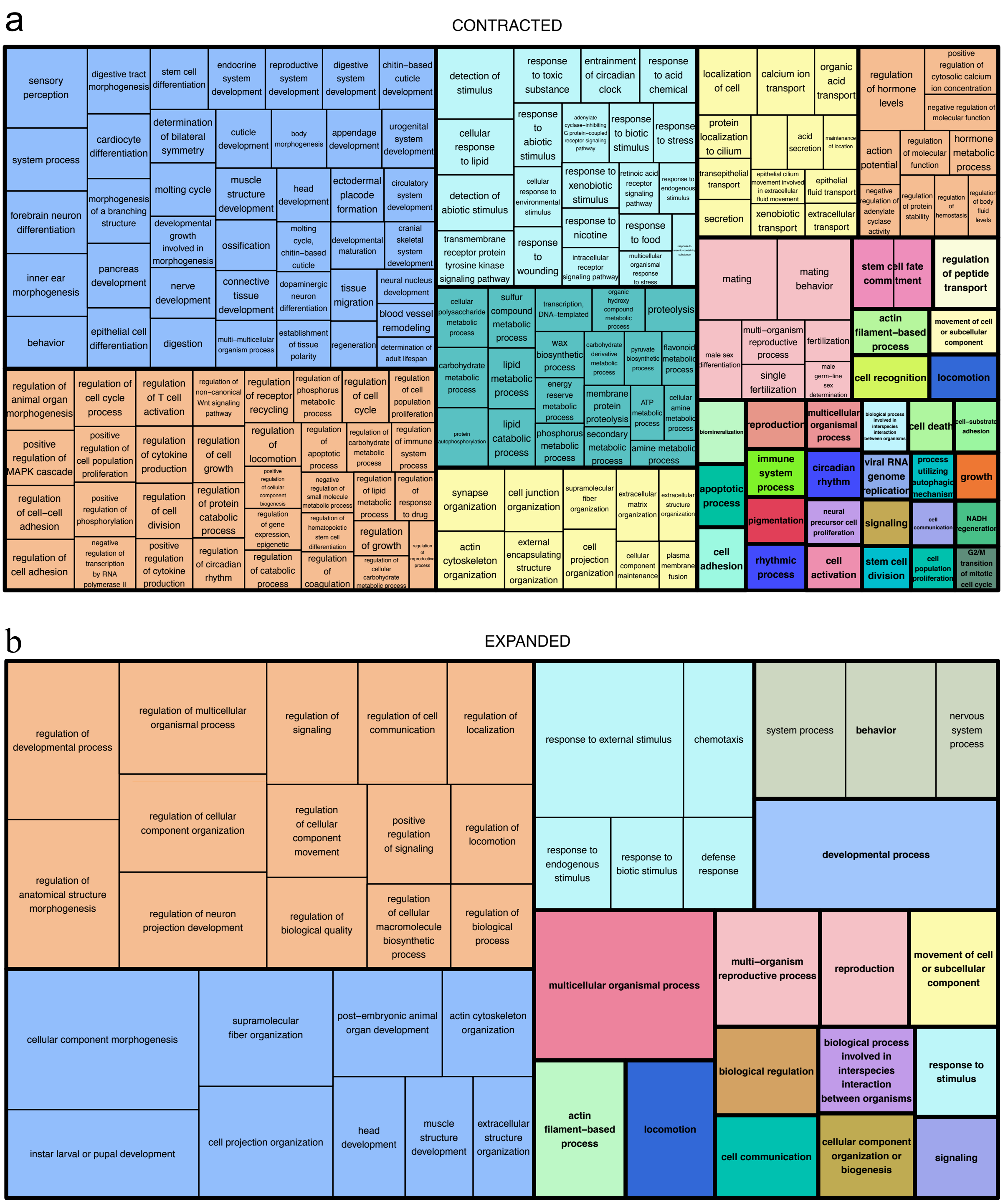

Supplementary Figure 1. Treemaps indicating the significantly overrepresented biological processes in the contracted (**a**), expanded (**b**) orthogroups in the most recent common ancestor of the tribe Bidessini obtained with a gene ontology enrichment. The size of the square is scaled to the p-value.

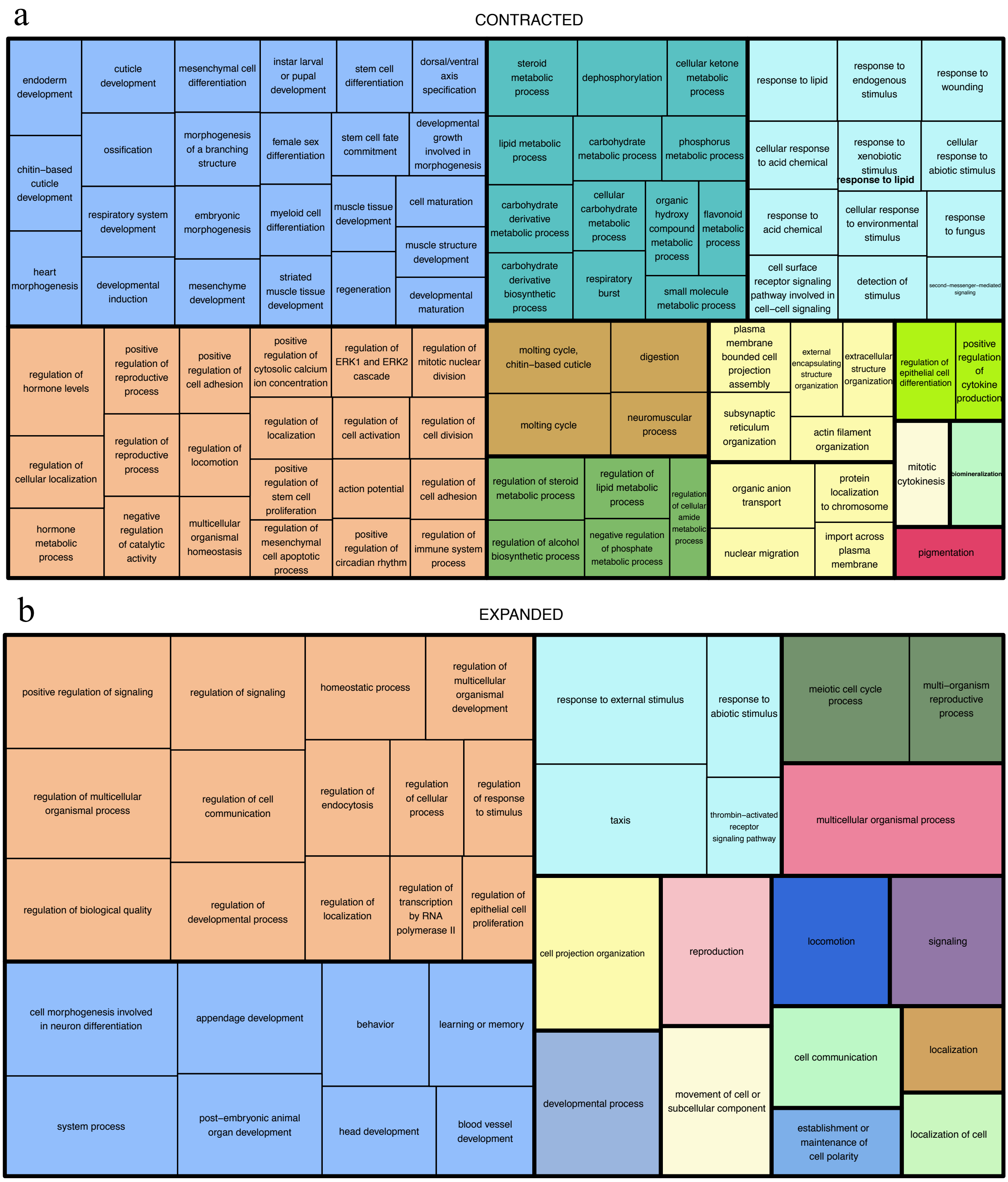

Supplementary Figure 2. Treemaps indicating the significantly overrepresented biological processes in the contracted (**a**), expanded (**b**) orthogroups in the most recent common ancestor of the tribe Hydroporini obtained with a gene ontology enrichment. The size of the square is scaled to the p-value.

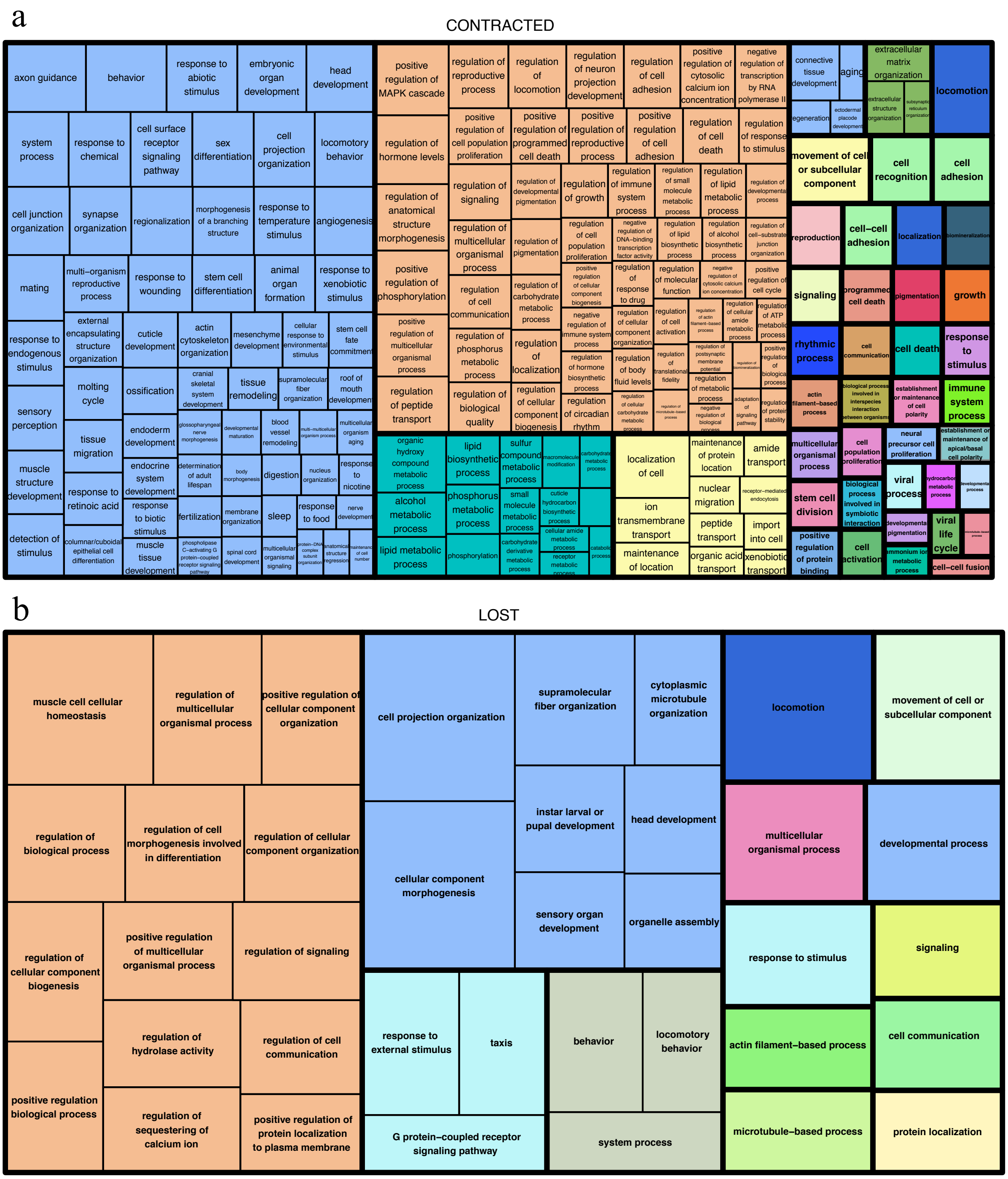

Supplementary Figure 3. Treemaps indicating the significantly overrepresented biological processes in the contracted (**a**) and lost (**b**) orthogroups in the most recent common ancestor of the tribe Leptodirini obtained with a gene ontology enrichment. The size of the square is scaled to the p-value.

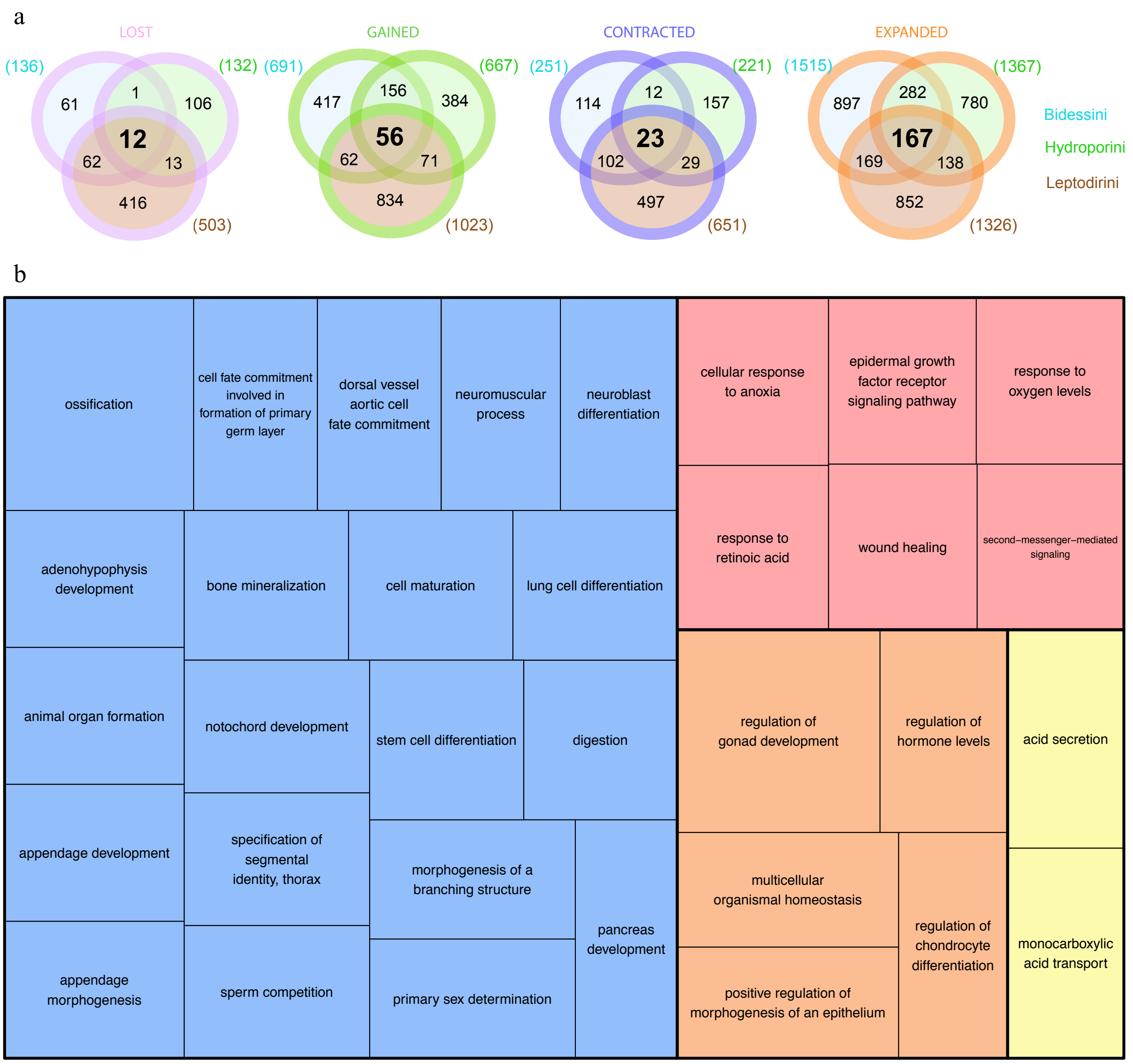

Supplementary Figure 4. Parallel evolution among the most recent common ancestors (MCRA) of the tribes Bidessini, Hydroporini and Leptodirini. **a** Venn diagrams indicating the total orthogroups with parallel evolution in the MRCA of the three tribes. **b** Treemap indicating the significantly overrepresented biological processes in the orthogroups parallelly contracted among the three MRCAs.

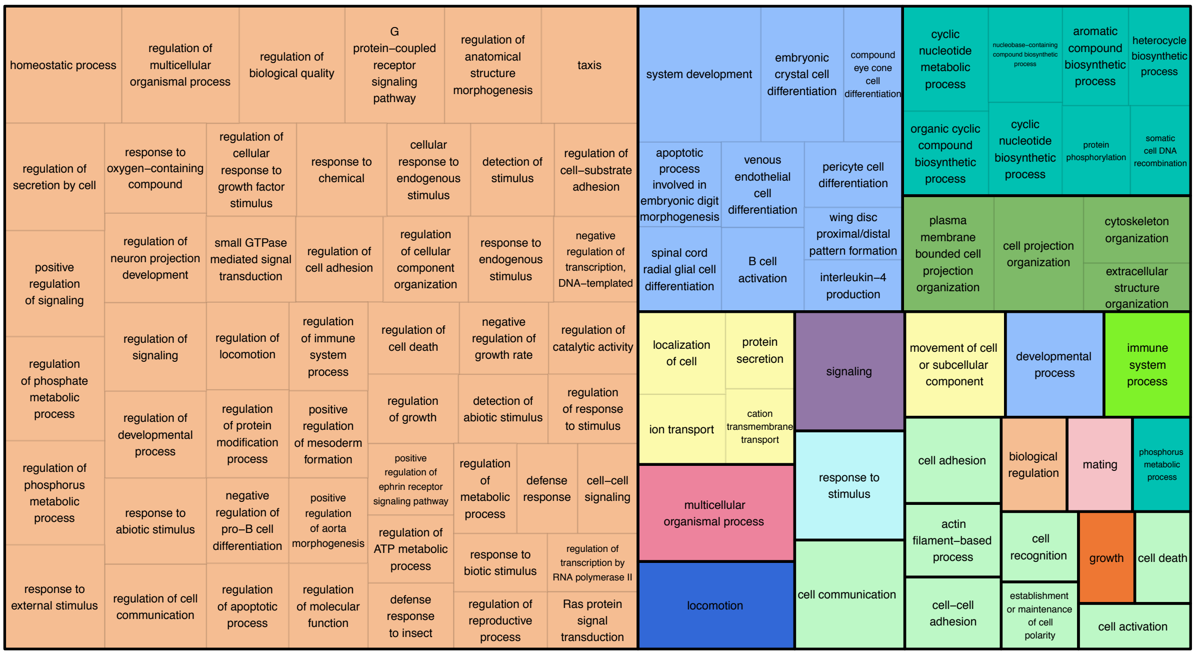

Supplementary Figure 5. Treemaps indicating the significantly overrepresented biological processes in the parallelly expanded orthogroups in the most recent common ancestor of the highly modified lineages of the tribe Leptodirini.

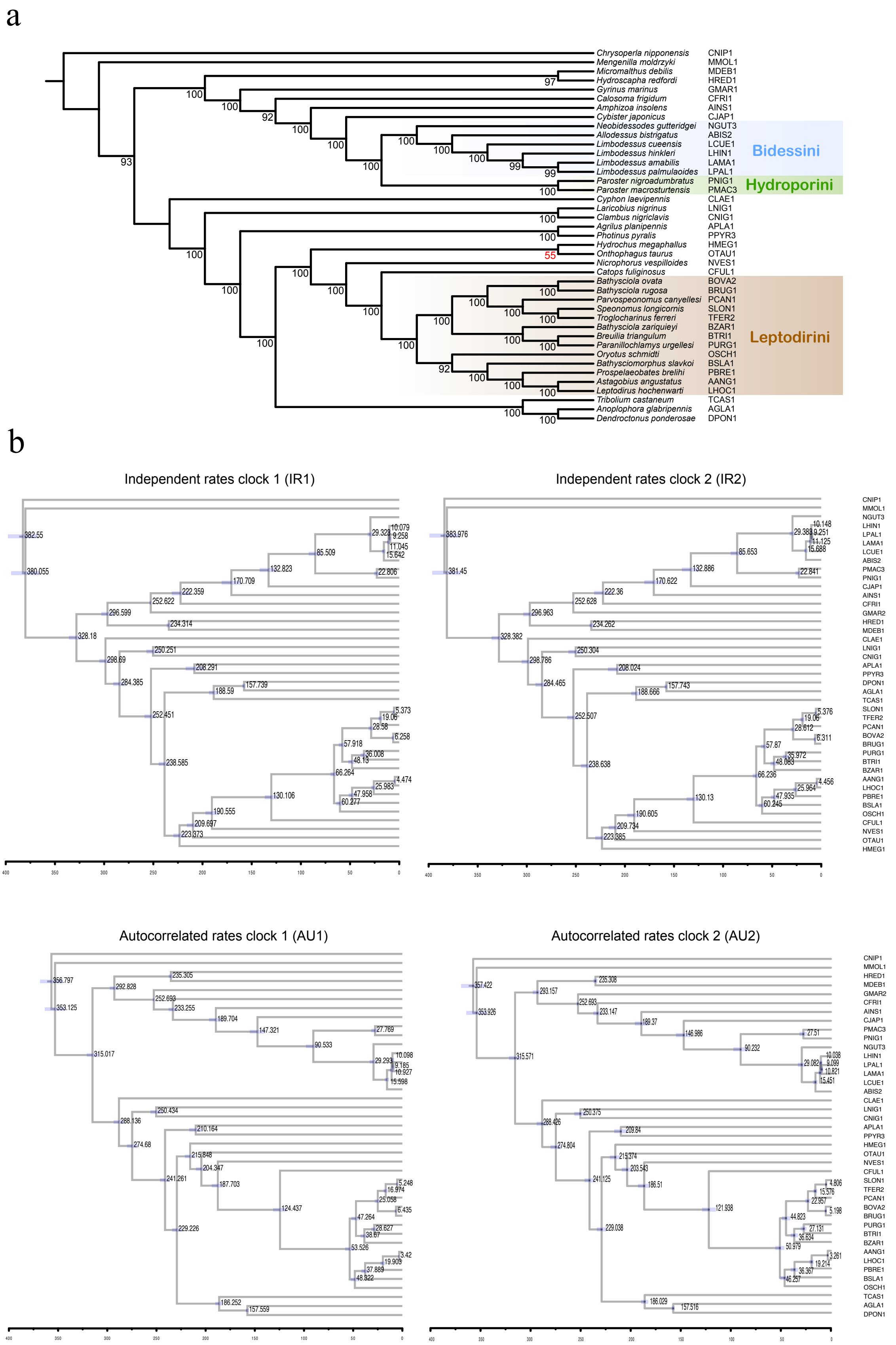

Supplementary Figure 6. a Maximum-likelihood phylogenetic tree inferred with 232 orthogroups. **b** Time-calibrated trees indicating the different time divergence estimations obtained with MCMCtree under different molecular clocks and alternative runs.
