# Supplementary material for: Parallel and convergent genomic changes underlie independent subterranean colonization across beetles
Source: Nat Commun. 2023 Jun 29;14:3842. doi: 10.1038/s41467-023-39603-1 (PMC10310748; doi:10.1038/s41467-023-39603-1)
Supplement: Supplementary file 3 — Description of Additional Supplementary Files [file 41467_2023_39603_MOESM3_ESM.pdf]

Description of Additional Supplementary Files

Supplementary Data 1:

Gene ontology (GO) similarity matrices for the exclusively contracted and expanded orthogroups (OGs), results of the affinity clustering approach and reduced GO terms for each functional cluster (i.e. Fig 4) and for the parallelly contracted and expanded OGs (i.e. Fig. 3) including the annotation results of OGs related to compound eye development that are expanded/contracted in parallel.

Supplementary Data 2:

Functional convergence (FC) results obtained with different sets of exclusively expanded and contracted orthogroups (OGs). Total exclusively contracted and expanded orthogroups (OGs) and number of functional clusters obtained through different comparisons. The FC ratio indicates the average number of OGs per functional cluster.

Supplementary Data 3:

Results of analysis of positive selection in highly modified cave lineages (HML) for the orthogroups expanded in both lineages (L1 and L3). Percentage of branches under positive selection is indicated for each orthogroup in each lineage. Functional annotation is included for each orthogroup when available (PFAM, KEGG_ko, KEGG_Pathway, GO, etc.).

Supplementary Data 4:

Information of the newly generated data regarding the collected species and RNA-seq data preprocessing results. After filtering (AF) results obtained with fastp are shown including the phred (base calling accuracy) values.

Supplementary Data 5:

Public data information and completeness score based on the obtained BUSCO results.

Supplementary Data 6:

Total number of orthogroups per species, corrected after the filtering with PRECUAL, and best-fitting model used for each phylogenetic inference obtained with ModelFinder.

Supplementary Data 7:

Results of the BadiRate analysis including the models comparison (i.e K indicates the number of parameters), significant OGs lists and their turnover rates (i.e. subterranean rates, SR; aquatic rates, AR; terrestrial rates, TR) for each species set and reconstructed evolutionary dynamics in the branches of interest.

Supplementary Data 8:

Orthogroup (OG) lists for each branch of interest, gene ontology (GO) annotation percentage and significantly enriched GO terms, including the most recent common ancestors (MRCA) of each tribe, the underground colonization events in Bidessini (B1, B2) Hydroporini (H1) and Leptodirini (L1, L2, L3) and the highly modified Leptodirini lineages (HML).
