# Supplementary material for: Parallel and convergent genomic changes underlie independent subterranean colonization across beetles
Source: Nat Commun. 2023 Jun 29;14:3842. doi: 10.1038/s41467-023-39603-1 (PMC10310748; doi:10.1038/s41467-023-39603-1)
Supplement: Supplementary file 12 — Reporting Summary [file 41467_2023_39603_MOESM12_ESM.pdf]

nature portfolio

Corresponding author(s): Rosa Fernández

Last updated by author(s): 8th June 2023

Reporting Summary

Nature Portfolio wishes to improve the reproducibility of the work that we publish. This form provides structure for consistency and transparency in reporting. For further information on Nature Portfolio policies, see our Editorial Policies and the Editorial Policy Checklist.

Statistics

For all statistical analyses, confirm that the following items are present in the figure legend, table legend, main text, or Methods section.

n/a	Confirmed	
☐	☒	The exact sample size (*n*) for each experimental group/condition, given as a discrete number and unit of measurement
☐	☒	A statement on whether measurements were taken from distinct samples or whether the same sample was measured repeatedly
☐	☒	The statistical test(s) used AND whether they are one- or two-sided<br>*Only common tests should be described solely by name; describe more complex techniques in the Methods section.*
☐	☒	A description of all covariates tested
☐	☒	A description of any assumptions or corrections, such as tests of normality and adjustment for multiple comparisons
☐	☒	A full description of the statistical parameters including central tendency (e.g. means) or other basic estimates (e.g. regression coefficient) AND variation (e.g. standard deviation) or associated estimates of uncertainty (e.g. confidence intervals)
☐	☒	For null hypothesis testing, the test statistic (e.g. *F*, *t*, *r*) with confidence intervals, effect sizes, degrees of freedom and *P* value noted<br>*Give P values as exact values whenever suitable.*
☐	☒	For Bayesian analysis, information on the choice of priors and Markov chain Monte Carlo settings
☐	☒	For hierarchical and complex designs, identification of the appropriate level for tests and full reporting of outcomes
☒	☐	Estimates of effect sizes (e.g. Cohen's *d*, Pearson's *r*), indicating how they were calculated

Our web collection on statistics for biologists contains articles on many of the points above.

Software and code

Policy information about availability of computer code

Data collection	We did not use any software for collecting data.
Data analysis	For the data processing workflow we used fastp v.0.20.0 to clean raw RNA-seq data, Trinity v2.11 to assembly clean data, TransDecoder v.5.5.0 to predict candidate coding regions, BlobTools2 to filter contaminant sequences, DIAMOND to annotate protein sequences prior to decontamination, Iso-Seq v.3.1.2 to process PacBio raw reads, ANGEL to predict open reading frames for the PacBio data, CD-HIT v.4.8.1 to cluster highly identical sequences, BUSCO v4.1.4 to assess the completeness of the gene content for the processed genomes and transcriptomes as a quality check measure.<br>For the phylogenomic analysis workflow we used OrthoFinder v.2.5.1 to infer orthologous groups, PREQUAL to filter bad quality sequences prior to the alignment, MAFFT v. 7.407 to align amino acid sequences, FastTree v.2.1.1 to perform maximum-likelihood phylogenetic inferences, ModelFinder to estimate the best model to perform phylogenetic inferences, IQTREE v.1.6.12 to perform maximum-likelihood phylogenetic inferences, PhyloPyPruner v.1.2.4 to filter short sequences and prune paralogs in given gene trees, GeneSortR to quantify phylogenetic usefulness and rank candidate orthogroups to be used in the molecular dating approach, MCMCtree to infer time-calibrated trees, Tracer v.1.7.1 to supervise different molecular clocks and alternative runs implemented in MCMCtree, iTOLv.6 to visualize and render the phylogenetic trees, BadiRate v.1.35 to explore gene family evolutionary dynamics with a maximum-likelihood approach, eggNOG-mapper v.2.1.6 to annotate amino acid sequences though an orthology based approach, GOEnrichment v.2.0 to perform gene ontology (GO) enrichment analysis, REVIGO to visualize and summarize the GO enrichment results, GOSemSim v.3.16 to calculate pairwise semantic similarity measures on lists of GO terms generating a GO similarity matrix, apcluster v.1.4.10 to perform an affinity propagation clustering on the GO similarity matrix, ComplexHeatmap v.3.15 and ggplot2 to plot the GO clustering results, GOexploreR v.1.2.6 and rrvgo v.1.8.1 to process and summarize long lists of GO terms based on their hierarchy and semantic similarity. These last four R packages were implemented with Rstudio v.4.2.1.<br>We used some custom bash and python scripts to process raw data following the approach used in Fernandez et al. (2022):

"fetch_longest_iso.py" and "remove_isoforms_proteome.sh" to filter the longest isoform per gene for transcriptomes and proteomes respectively in order to obtain candidate gene sets for further analyses and "blobtools.sh" and "extract_phyla_for_blobtools.py" for the decontamination protocol. Moreover, we used the custom R script "get_badirate_gains_losses.R" to process information about gene family expansion and contraction from the raw output of BadiRate.

For manuscripts utilizing custom algorithms or software that are central to the research but not yet described in published literature, software must be made available to editors and reviewers. We strongly encourage code deposition in a community repository (e.g. GitHub). See the Nature Portfolio guidelines for submitting code & software for further information.

Data

Policy information about availability of data

All manuscripts must include a data availability statement. This statement should provide the following information, where applicable:

- Accession codes, unique identifiers, or web links for publicly available datasets
- A description of any restrictions on data availability
- For clinical datasets or third party data, please ensure that the statement adheres to our policy

Raw reads have been deposited in the National Center for Biotechnology Information (NCBI; BioProject accession number PRJNA902350). All processed data and custom scripts have been deposited in github (https://github.com/MetazoaPhylogenomicsLab/Balart_Garcia_et_al_gene_repertoire_evolution_subterranean_coleoptera).

Human research participants

Policy information about studies involving human research participants and Sex and Gender in Research.

Reporting on sex and gender	Our research is not on humans.
Population characteristics	See above
Recruitment	See above
Ethics oversight	See above

Note that full information on the approval of the study protocol must also be provided in the manuscript.

Field-specific reporting

Please select the one below that is the best fit for your research. If you are not sure, read the appropriate sections before making your selection.

☐ Life sciences ☐ Behavioural & social sciences ☒ Ecological, evolutionary & environmental sciences

For a reference copy of the document with all sections, see nature.com/documents/nr-reporting-summary-flat.pdf

Life sciences study design

All studies must disclose on these points even when the disclosure is negative.

Sample size	*Describe how sample size was determined, detailing any statistical methods used to predetermine sample size OR if no sample-size calculation was performed, describe how sample sizes were chosen and provide a rationale for why these sample sizes are sufficient.*
Data exclusions	*Describe any data exclusions. If no data were excluded from the analyses, state so OR if data were excluded, describe the exclusions and the rationale behind them, indicating whether exclusion criteria were pre-established.*
Replication	*Describe the measures taken to verify the reproducibility of the experimental findings. If all attempts at replication were successful, confirm this OR if there are any findings that were not replicated or cannot be reproduced, note this and describe why.*
Randomization	*Describe how samples/organisms/participants were allocated into experimental groups. If allocation was not random, describe how covariates were controlled OR if this is not relevant to your study, explain why.*
Blinding	*Describe whether the investigators were blinded to group allocation during data collection and/or analysis. If blinding was not possible, describe why OR explain why blinding was not relevant to your study.*

Behavioural & social sciences study design

All studies must disclose on these points even when the disclosure is negative.

Study description	*Briefly describe the study type including whether data are quantitative, qualitative, or mixed-methods (e.g. qualitative cross-sectional, quantitative experimental, mixed-methods case study).*
Research sample	*State the research sample (e.g. Harvard university undergraduates, villagers in rural India) and provide relevant demographic information (e.g. age, sex) and indicate whether the sample is representative. Provide a rationale for the study sample chosen. For studies involving existing datasets, please describe the dataset and source.*
Sampling strategy	*Describe the sampling procedure (e.g. random, snowball, stratified, convenience). Describe the statistical methods that were used to predetermine sample size OR if no sample-size calculation was performed, describe how sample sizes were chosen and provide a rationale for why these sample sizes are sufficient. For qualitative data, please indicate whether data saturation was considered, and what criteria were used to decide that no further sampling was needed.*
Data collection	*Provide details about the data collection procedure, including the instruments or devices used to record the data (e.g. pen and paper, computer, eye tracker, video or audio equipment) whether anyone was present besides the participant(s) and the researcher, and whether the researcher was blind to experimental condition and/or the study hypothesis during data collection.*
Timing	*Indicate the start and stop dates of data collection. If there is a gap between collection periods, state the dates for each sample cohort.*
Data exclusions	*If no data were excluded from the analyses, state so OR if data were excluded, provide the exact number of exclusions and the rationale behind them, indicating whether exclusion criteria were pre-established.*
Non-participation	*State how many participants dropped out/declined participation and the reason(s) given OR provide response rate OR state that no participants dropped out/declined participation.*
Randomization	*If participants were not allocated into experimental groups, state so OR describe how participants were allocated to groups, and if allocation was not random, describe how covariates were controlled.*

Ecological, evolutionary & environmental sciences study design

All studies must disclose on these points even when the disclosure is negative.

Study description	We explored the molecular underpinnings of adaptations to life in caves in Coleoptera using a genome-wide phylogenomic approach. To do so, we investigate and characterize evolutionary dynamics of the gene repertoire across surface-dwelling and subterranean species that represent multiple independent underground colonizations both in terrestrial and aquatic beetle lineages. Our results demonstrate that genomic exaptation prior to the colonization of subterranean habitats was key to facilitating these transitions in terrestrial and aquatic lineages, and that both parallel and convergent evolution paved the way to adapting to life in caves across the Coleoptera.
Research sample	We collected surface-dwelling and subterranean species of the tribes Leptodirini, that have a terrestrial ecology, and species of the tribes Bidessini and Hydroporini, that have an aquatic ecology. These three tribes correspond to two different subfamilies of beetles (Cholevinae and Hydroporinae) that diverged more than 300 Mya. Each tribe include several lineages that colonized independently terrestrial and aquatic subterranean habitats, representing ideal systems to explore parallel evolution and functional convergence in subterranean fauna from a macroevolutionary perspective. We selected 21 species resenting at least six independent underground colonization events, including surface-dwelling and subterranean ecologies. Moreover we included 1 species of the tribe Catopini, representing a closely related lineage to Leptodirini.
Sampling strategy	We sampled between 15 and 2 specimens per species, depending on the availability and to ensure a efficient RNA isolation.
Data collection	We generated data for a total of 21 species, including 12 from the tribe Leptodirini, 1 from the tribe Catopini (i.e. a closely related tribe to Leptodirini), 2 from the tribe Hydroporini and 6 from the tribe Bidessini. Data collection for species of the tribe Leptodirini was in charge of Pau Balart-Garcia, Slavko Polak and Ignacio Ribera who participated in the sampling procedures such as selecting the species based on their phylogenetic relationships, planning the field trips and collecting the specimens. Wet lab procedures were done by Pau Balart-Garcia. Data generation for species of the tribes Bidessini and Hydroporini species was in charge of Steven J. B. Cooper and wet lab procedures were done by Tessa M. Bradford and Perry G. Beasley-Hall.<br>Moreover, we collected publicly available transcriptomic and genomic raw data of other Coleoptera species and two outgroups (i.e. Strepsiptera and Neuroptera) in order to include a broader phylogenetic context to study gene repertoire evolution. This part was in charge of Pau Balart-Garcia, who selected and processed all the raw data.
Timing and spatial scale	Data was collected between 2018 and 2020 for several parallel projects. Once we collected all the data we proceeded with the processing analyses in order to follow the same strategy for all the data sets.
Data exclusions	During the collection of publicly available data we discarded several genomes and transcriptomes that did not pass the final quality check, which consist of estimating the completeness of the gene content based on the sets of benchmarking universal single-copy

	orthologs with BUSCO. The data sets with less than 80 % of complete plus fragmented genes were excluded.
Reproducibility	To ensure reproducibility we used the same data processing protocols for all the data sets.
Randomization	We processed the samples that we randomly collected in the field and ensuring that they correspond to adult specimens of the same species.
Blinding	We do not work on humans.

Did the study involve field work? ☒ Yes ☐ No

Field work, collection and transport

Field conditions	The surface-dwelling species of the tribe Leptodirini and a species of the tribe Catopini (Catops fuliginosus) were collected in forest-litter using an entomological litter reducer and a metallic sieve of 3mm aperture. The subterranean species were captured manually in caves using an entomological aspirator. Temperature and humidity is constant in caves, any season is suitable to collect cave-dwelling species. Forest-litter species were collected during optimal climatic conditions for an effective sampling, specially at the end of spring or autumn to ensure a moderate environmental humidity (i.e. not water saturated, frozen or dry environment where animals are not present). The surface-dwelling species of the tribes Bidessini and Hydroporini were captured using aquatic nets in shallow still water and temporary pools that tend to be formed during the rainy seasons, specially in spring. Groundwater-monitoring boreholes were used to access the habitat of the subterranean species of Bidessini and Hydroporini and specimens were captured with a mesh net attached to a fishing rod. Likely to caves, the environmental conditions of groundwater habitats are stable and there is not a strong seasonality influencing specimen collection. When the localities were remote, we kept specimens in RNA-later and cold conditions to reduce risk of RNA degradation. Otherwise, specimens were kept alive in thermo boxes with moss to keep the humidity (terrestrial beetles) or tubes with the same water where they were found (aquatic beetles). Samples were transported in dark and constant temperature simulating the conditions of their habitat.
Location	Samples were collected in 21 different localities with the following coordinates: Breuilia triangulum (43.4016, -4.8214), Bathysciola rugosa (42.9698, -1.9445), Parvospeonomus canyellesi (41.7444, 2.4850), Astagobius angustatus (45.8215, 14.0676), Prospelaeobates brelihi (45.6171, 14.3426), Catops fuliginosus (41.3931, 1.91102), Leptodirus hochenwartii (45.6970, 14.1299), Troglocharinus ferreri (41.4019, 1.88525), Bathysciola zariquieyi (41.4150, 2.09765), Paranillochlamys urgellesi (40.6353, 0.56492), Bathysciola ovata (42.7659, 0.60492), Oryotus schmidti (45.8215, 14.0676), Bathysciomorphus slavkoi (45.6611, 14.2230), Limbodessus cueensis (-27.2696, 117.9896), Limbodessus hinkleri (-26.8793, 120.1635), Allodessus bistrigatus (-34.6889, 138.8933), Limbodessus palmulaoides (-28.3983, 122.2038), Paroster nigroadumbratus (-34.6889, 138.8933), Paroster macrosturtensis (-28.7155, 120.8931), Neobidessodes gutteridgei (-25.2786, 119.1834), Limbodessus amabilis (-34.99, 138.82) (see Supplementary Table 3 for detailed information of the sampling sites). Cave-dwelling species with a terrestrial ecology were collected in deep parts of the caves (more than 50 meters from the entrance in absolute darkness, what is known as the "deep subterranean environment"). Forest-litter specimens were collected sifting the first 10-20 cm of leaf-litter. Aquatic beetles living in surface habitats were collected in shallow water (1-50cm) and groundwater species were collected by sampling the water column which vary between 1 to 15m.
Access & import/export	Specimens were collected under permits 35601-58/2020-5 (Agencija RS za okolje, Ministrstvo za okolje in prostor, Republika Slovenija) and SF010263 (Department of Parks and Wildlife, WA)
Disturbance	No remarkable disturbance to declare, samples were collected manually using selective methods that minimize the environmental impact.

Reporting for specific materials, systems and methods

We require information from authors about some types of materials, experimental systems and methods used in many studies. Here, indicate whether each material, system or method listed is relevant to your study. If you are not sure if a list item applies to your research, read the appropriate section before selecting a response.

Materials & experimental systems

n/a	Involved in the study	
☐	☐	Antibodies
☐	☐	Eukaryotic cell lines
☐	☐	Palaeontology and archaeology
☐	☒	Animals and other organisms
☐	☐	Clinical data
☐	☐	Dual use research of concern

Methods

n/a	Involved in the study	
☐	☐	ChIP-seq
☐	☐	Flow cytometry
☐	☐	MRI-based neuroimaging

Antibodies

Antibodies used	*Describe all antibodies used in the study; as applicable, provide supplier name, catalog number, clone name, and lot number.*
Validation	*Describe the validation of each primary antibody for the species and application, noting any validation statements on the manufacturer's website, relevant citations, antibody profiles in online databases, or data provided in the manuscript.*

Eukaryotic cell lines

Policy information about cell lines and Sex and Gender in Research

Cell line source(s)	*State the source of each cell line used and the sex of all primary cell lines and cells derived from human participants or vertebrate models.*
Authentication	*Describe the authentication procedures for each cell line used OR declare that none of the cell lines used were authenticated.*
Mycoplasma contamination	*Confirm that all cell lines tested negative for mycoplasma contamination OR describe the results of the testing for mycoplasma contamination OR declare that the cell lines were not tested for mycoplasma contamination.*
Commonly misidentified lines (See ICLAC register)	*Name any commonly misidentified cell lines used in the study and provide a rationale for their use.*

Palaeontology and Archaeology

Specimen provenance	*Provide provenance information for specimens and describe permits that were obtained for the work (including the name of the issuing authority, the date of issue, and any identifying information). Permits should encompass collection and, where applicable, export.*
Specimen deposition	*Indicate where the specimens have been deposited to permit free access by other researchers.*
Dating methods	*If new dates are provided, describe how they were obtained (e.g. collection, storage, sample pretreatment and measurement), where they were obtained (i.e. lab name), the calibration program and the protocol for quality assurance OR state that no new dates are provided.*

☐ Tick this box to confirm that the raw and calibrated dates are available in the paper or in Supplementary Information.

Ethics oversight	*Identify the organization(s) that approved or provided guidance on the study protocol, OR state that no ethical approval or guidance was required and explain why not.*

Note that full information on the approval of the study protocol must also be provided in the manuscript.

Animals and other research organisms

Policy information about studies involving animals; ARRIVE guidelines recommended for reporting animal research, and Sex and Gender in Research

Laboratory animals	The study did not include laboratory animals.
Wild animals	The surface-dwelling species of the tribe Leptodirini (Bathysciola rugosa, Bathysciola zariquieyi, Bathysciola ovata) and a species of the tribe Catopini (Catops fuliginosus) were collected in forest-litter using an entomological litter reducer and a metallic sieve of 3mm aperture. The subterranean species (Breuilia triangulum, Parvospeonomus canyellesi, Astagobius angustatus, Prospelaeobates brelihi, Leptodirus hochenwartii, Troglocharinus ferreri, Paranillochlamys urgellesi, Oryotus schmidti, Bathysciomorphus slavkoi) were captured manually in caves using an entomological aspirator. The surface-dwelling species of the tribes Bidessini (Allodessus bistrigatus and Limbodessus amabilis) and Hydroporini (Paroster nigroadumbratus) were captured using aquatic nets in shallow still water and temporary pools that tend to be formed in spring. Groundwater-monitoring boreholes were used to access the habitat of the subterranean species of Bidessini (Neobidessodes gutteridgei, Limbodessus cueensis, Limbodessus hinkleri, Limbodessus palmulaoides) and Hydroporini (Paroster macrosturtensis) and specimens were captured with a mesh net attached to a fishing rod. When the localities were remote, we kept specimens in RNA-later and cold conditions to reduce risk of RNA degradation. Otherwise, specimens were kept alive in thermo boxes with moss to keep the humidity (terrestrial beetles) or tubes with the same water where they were found (aquatic beetles). Samples were transported in dark and constant temperature simulating the conditions of their habitat. Specimens were identified and killed flash-frozen with liquid nitrogen once we arrived to the laboratory. Specimens were killed in order to extract RNA and generate RNA-seq data.
Reporting on sex	Not relevant for this study.
Field-collected samples	Specimens were stored at -80ºC immediately when arriving to the laboratory.
Ethics oversight	To our knowledge there is not official ethics protocol for wild insects.

Note that full information on the approval of the study protocol must also be provided in the manuscript.

Clinical data

Policy information about clinical studies
All manuscripts should comply with the ICMJE guidelines for publication of clinical research and a completed CONSORT checklist must be included with all submissions.

Clinical trial registration	*Provide the trial registration number from ClinicalTrials.gov or an equivalent agency.*
Study protocol	*Note where the full trial protocol can be accessed OR if not available, explain why.*
Data collection	*Describe the settings and locales of data collection, noting the time periods of recruitment and data collection.*
Outcomes	*Describe how you pre-defined primary and secondary outcome measures and how you assessed these measures.*

Dual use research of concern

Policy information about dual use research of concern

Hazards

Could the accidental, deliberate or reckless misuse of agents or technologies generated in the work, or the application of information presented in the manuscript, pose a threat to:

No	Yes	
☒	☐	Public health
☒	☐	National security
☒	☐	Crops and/or livestock
☒	☐	Ecosystems
☒	☐	Any other significant area

Experiments of concern

Does the work involve any of these experiments of concern:

No	Yes	
☒	☐	Demonstrate how to render a vaccine ineffective
☒	☐	Confer resistance to therapeutically useful antibiotics or antiviral agents
☒	☐	Enhance the virulence of a pathogen or render a nonpathogen virulent
☒	☐	Increase transmissibility of a pathogen
☒	☐	Alter the host range of a pathogen
☒	☐	Enable evasion of diagnostic/detection modalities
☒	☐	Enable the weaponization of a biological agent or toxin
☒	☐	Any other potentially harmful combination of experiments and agents

ChIP-seq

Data deposition

☐ Confirm that both raw and final processed data have been deposited in a public database such as GEO.

☐ Confirm that you have deposited or provided access to graph files (e.g. BED files) for the called peaks.

Data access links<br>*May remain private before publication.*	*For "Initial submission" or "Revised version" documents, provide reviewer access links. For your "Final submission" document, provide a link to the deposited data.*
Files in database submission	*Provide a list of all files available in the database submission.*
Genome browser session<br>(e.g. UCSC)	*Provide a link to an anonymized genome browser session for "Initial submission" and "Revised version" documents only, to enable peer review. Write "no longer applicable" for "Final submission" documents.*

Methodology

Replicates	*Describe the experimental replicates, specifying number, type and replicate agreement.*

Sequencing depth | *Describe the sequencing depth for each experiment, providing the total number of reads, uniquely mapped reads, length of reads and whether they were paired- or single-end.*

Antibodies | *Describe the antibodies used for the ChIP-seq experiments; as applicable, provide supplier name, catalog number, clone name, and lot number.*

Peak calling parameters | *Specify the command line program and parameters used for read mapping and peak calling, including the ChIP, control and index files used.*

Data quality | *Describe the methods used to ensure data quality in full detail, including how many peaks are at FDR 5% and above 5-fold enrichment.*

Software | *Describe the software used to collect and analyze the ChIP-seq data. For custom code that has been deposited into a community repository, provide accession details.*

Flow Cytometry

Plots

Confirm that:

- ☐ The axis labels state the marker and fluorochrome used (e.g. CD4-FITC).
- ☐ The axis scales are clearly visible. Include numbers along axes only for bottom left plot of group (a 'group' is an analysis of identical markers).
- ☐ All plots are contour plots with outliers or pseudocolor plots.
- ☐ A numerical value for number of cells or percentage (with statistics) is provided.

Methodology

Sample preparation | *Describe the sample preparation, detailing the biological source of the cells and any tissue processing steps used.*

Instrument | *Identify the instrument used for data collection, specifying make and model number.*

Software | *Describe the software used to collect and analyze the flow cytometry data. For custom code that has been deposited into a community repository, provide accession details.*

Cell population abundance | *Describe the abundance of the relevant cell populations within post-sort fractions, providing details on the purity of the samples and how it was determined.*

Gating strategy | *Describe the gating strategy used for all relevant experiments, specifying the preliminary FSC/SSC gates of the starting cell population, indicating where boundaries between "positive" and "negative" staining cell populations are defined.*

☐ Tick this box to confirm that a figure exemplifying the gating strategy is provided in the Supplementary Information.

Magnetic resonance imaging

Experimental design

Design type | *Indicate task or resting state; event-related or block design.*

Design specifications | *Specify the number of blocks, trials or experimental units per session and/or subject, and specify the length of each trial or block (if trials are blocked) and interval between trials.*

Behavioral performance measures | *State number and/or type of variables recorded (e.g. correct button press, response time) and what statistics were used to establish that the subjects were performing the task as expected (e.g. mean, range, and/or standard deviation across subjects).*

Acquisition

Imaging type(s) | *Specify: functional, structural, diffusion, perfusion.*

Field strength | *Specify in Tesla*

Sequence & imaging parameters | *Specify the pulse sequence type (gradient echo, spin echo, etc.), imaging type (EPI, spiral, etc.), field of view, matrix size, slice thickness, orientation and TE/TR/flip angle.*

Area of acquisition | *State whether a whole brain scan was used OR define the area of acquisition, describing how the region was determined.*

Diffusion MRI ☐ Used ☐ Not used

Preprocessing

Preprocessing software	*Provide detail on software version and revision number and on specific parameters (model/functions, brain extraction, segmentation, smoothing kernel size, etc.).*
Normalization	*If data were normalized/standardized, describe the approach(es): specify linear or non-linear and define image types used for transformation OR indicate that data were not normalized and explain rationale for lack of normalization.*
Normalization template	*Describe the template used for normalization/transformation, specifying subject space or group standardized space (e.g. original Talairach, MNI305, ICBM152) OR indicate that the data were not normalized.*
Noise and artifact removal	*Describe your procedure(s) for artifact and structured noise removal, specifying motion parameters, tissue signals and physiological signals (heart rate, respiration).*
Volume censoring	*Define your software and/or method and criteria for volume censoring, and state the extent of such censoring.*

Statistical modeling & inference

Model type and settings	*Specify type (mass univariate, multivariate, RSA, predictive, etc.) and describe essential details of the model at the first and second levels (e.g. fixed, random or mixed effects; drift or auto-correlation).*
Effect(s) tested	*Define precise effect in terms of the task or stimulus conditions instead of psychological concepts and indicate whether ANOVA or factorial designs were used.*

Specify type of analysis: ☐ Whole brain ☐ ROI-based ☐ Both

Statistic type for inference (See Eklund et al. 2016)	*Specify voxel-wise or cluster-wise and report all relevant parameters for cluster-wise methods.*
Correction	*Describe the type of correction and how it is obtained for multiple comparisons (e.g. FWE, FDR, permutation or Monte Carlo).*

Models & analysis

n/a	Involved in the study
☐	☐ Functional and/or effective connectivity
☐	☐ Graph analysis
☐	☐ Multivariate modeling or predictive analysis

Functional and/or effective connectivity	*Report the measures of dependence used and the model details (e.g. Pearson correlation, partial correlation, mutual information).*
Graph analysis	*Report the dependent variable and connectivity measure, specifying weighted graph or binarized graph, subject- or group-level, and the global and/or node summaries used (e.g. clustering coefficient, efficiency, etc.).*
Multivariate modeling and predictive analysis	*Specify independent variables, features extraction and dimension reduction, model, training and evaluation metrics.*
